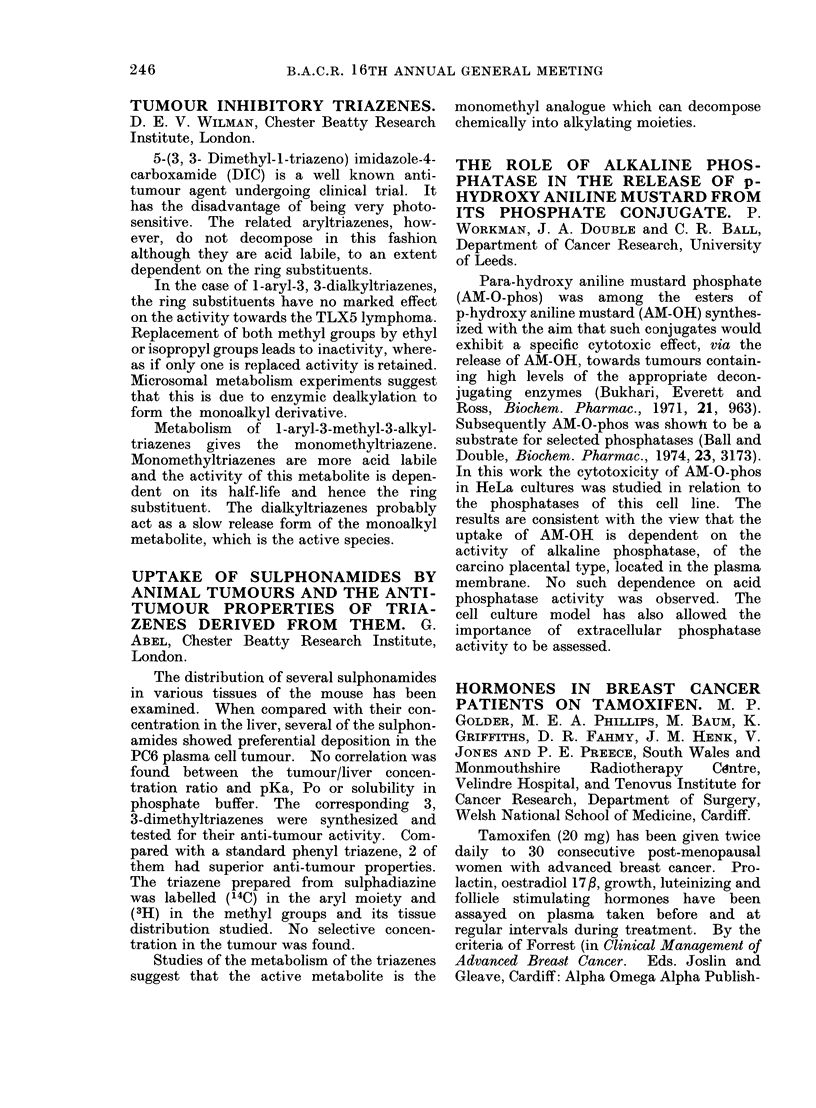# Proceedings: The role of alkaline phosphatase in the release of p-hydroxy aniline mustard from its phosphate conjugate.

**DOI:** 10.1038/bjc.1975.175

**Published:** 1975-08

**Authors:** P. Workman, J. A. Double, C. R. Ball


**THE ROLE OF ALKALINE PHOSPHATASE IN THE RELEASE OF p-HYDROXY ANILINE MUSTARD FROM ITS PHOSPHATE CONJUGATE.** P. WORKMAN, J. A. DOUBLE and C. R. BALL, Department of Cancer Research, University of Leeds.

Para-hydroxy aniline mustard phosphate (AM-O-phos) was among the esters of p-hydroxy aniline mustard (AM-OH) synthesized with the aim that such conjugates would exhibit a specific cytotoxic effect, *via* the release of AM-OH, towards tumours containing high levels of the appropriate deconjugating enzymes (Bukhari, Everett and Ross, *Biochem. Pharmac.*, 1971, **21**, 963). Subsequently AM-O-phos was shown to be a substrate for selected phosphatases (Ball and Double, *Biochem. Pharmac.*, 1974, **23**, 3173). In this work the cytotoxicity of AM-O-phos in HeLa cultures was studied in relation to the phosphatases of this cell line. The results are consistent with the view that the uptake of AM-OH is dependent on the activity of alkaline phosphatase, of the carcino placental type, located in the plasma membrane. No such dependence on acid phosphatase activity was observed. The cell culture model has also allowed the importance of extracellular phosphatase activity to be assessed.